# The chloroplast genome sequence of the green alga *Leptosira terrestris*: multiple losses of the inverted repeat and extensive genome rearrangements within the Trebouxiophyceae

**DOI:** 10.1186/1471-2164-8-213

**Published:** 2007-07-04

**Authors:** Jean-Charles de Cambiaire, Christian Otis, Monique Turmel, Claude Lemieux

**Affiliations:** 1 Département de biochimie et de microbiologie, Université Laval, Québec, Canada

## Abstract

**Background:**

In the Chlorophyta – the green algal phylum comprising the classes Prasinophyceae, Ulvophyceae, Trebouxiophyceae and Chlorophyceae – the chloroplast genome displays a highly variable architecture. While chlorophycean chloroplast DNAs (cpDNAs) deviate considerably from the ancestral pattern described for the prasinophyte *Nephroselmis olivacea*, the degree of remodelling sustained by the two ulvophyte cpDNAs completely sequenced to date is intermediate relative to those observed for chlorophycean and trebouxiophyte cpDNAs. *Chlorella vulgaris *(Chlorellales) is currently the only photosynthetic trebouxiophyte whose complete cpDNA sequence has been reported. To gain insights into the evolutionary trends of the chloroplast genome in the Trebouxiophyceae, we sequenced cpDNA from the filamentous alga *Leptosira terrestris *(Ctenocladales).

**Results:**

The 195,081-bp *Leptosira *chloroplast genome resembles the 150,613-bp *Chlorella *genome in lacking a large inverted repeat (IR) but differs greatly in gene order. Six of the conserved genes present in *Chlorella *cpDNA are missing from the *Leptosira *gene repertoire. The 106 conserved genes, four introns and 11 free standing open reading frames (ORFs) account for 48.3% of the genome sequence. This is the lowest gene density yet observed among chlorophyte cpDNAs. Contrary to the situation in *Chlorella *but similar to that in the chlorophycean *Scenedesmus obliquus*, the gene distribution is highly biased over the two DNA strands in *Leptosira*. Nine genes, compared to only three in *Chlorella*, have significantly expanded coding regions relative to their homologues in ancestral-type green algal cpDNAs. As observed in chlorophycean genomes, the *rpoB *gene is fragmented into two ORFs. Short repeats account for 5.1% of the *Leptosira *genome sequence and are present mainly in intergenic regions.

**Conclusion:**

Our results highlight the great plasticity of the chloroplast genome in the Trebouxiophyceae and indicate that the IR was lost on at least two separate occasions. The intriguing similarities of the derived features exhibited by *Leptosira *cpDNA and its chlorophycean counterparts suggest that the same evolutionary forces shaped the IR-lacking chloroplast genomes in these two algal lineages.

## Background

All chloroplasts of photosynthetic eukaryotes inherited from their cyanobacterial ancestors a reduced genome that encodes part of the genes essential for their biogenesis. The chloroplast genome has been studied in various algal lineages, particularly in the green algal/land plant lineage (Viridiplantae) for which the number of complete chloroplast DNA (cpDNA) sequences available in public databases increases steadily. Comparative analyses of the latter genome sequences highlight distinct evolutionary trends in the Streptophyta and the Chlorophyta. In the Streptophyta, the division comprising the green algae from the class Charophyceae and all land plants, the chloroplast genome shows remarkable conservation in overall structure, gene content, gene order and intron content [1,2]. In contrast, considerable fluidity in chloroplast genome organization is the hallmark of the Chlorophyta, the division comprising the four remaining green algal classes (Prasinophyceae, Ulvophyceae, Trebouxiophyceae and Chlorophyceae).

The chloroplast genomes from members of the Ulvophyceae (*Oltmannsiellopsis viridis *[3] and *Pseudendoclonium akinetum *[4]), Trebouxiophyceae (*Chlorella vulgaris *[5] and the non-photosynthetic alga *Helicosporidium sp*. [6]) and Chlorophyceae (*Stigeoclonium helveticum *[7], *Scenedesmus obliquus *[8] and *Chlamydomonas reinhardtii *[9]) display various patterns of divergence compared to the ancestral pattern described for a representative of the Prasinophyceae (*Nephroselmis olivacea *[10]). The cpDNA of this prasinophyte resembles most of its streptophyte homologues in displaying small and large single-copy (SSC and LSC) regions that are separated from one another by two identical inverted repeat regions (IR). Moreover, the set of genes encoded by each of these three genomic regions is almost identical in *Nephroselmis *and streptophyte cpDNAs. Moderate deviations from this ancestral-type architecture are seen in the two earliest-diverging lineages of the Ulvophyceae (the orders Oltmannsiellopsidales and Ulotrichales), supporting the hypothesis that a dozen genes were transferred from the LSC to the SSC region and that the rRNA operon in the IR was altered in orientation very early during the evolution of ulvophytes [3]. Although the cpDNAs of the chlorophycean green algae *Chlamydomonas *and *Scenedesmus *have also retained a quadripartite structure, the single-copy regions of these genomes differ extensively in gene content and both genomes deviate radically from the ancestral gene partitioning pattern [8]. The chloroplast genomes of *Chlorella *[5] and *Stigeoclonium *[7] as well as the plastid genome of *Helicosporidium *[6] lack an IR, indicating that this repeat was lost independently in the Trebouxiophyceae and Chlorophyceae. Considering that loss of the IR is often correlated with gene rearrangements [11,12], it is noteworthy that the IR-lacking cpDNA of the trebouxiophyte *Chlorella *retains an almost intact pattern of ancestral gene partitioning [3].

In *Helicosporidium *and the three lineages of chlorophycean green algae examined, remodelling of plastid/chloroplast genome architecture was accompanied by the formation of long blocks of consecutive genes on the same DNA strand [6-8,13]. For the *Stigeoclonium *and *Helicosporidium *genomes, the pattern of gene distribution between the two DNA strands was found to be correlated with a bias in base composition along each strand, allowing the identification of a potential origin of replication [6,7].

The cpDNAs of ulvophyte, trebouxiophyte and chlorophycean (UTC) green algae also underwent erosion of ancestral gene clusters, many gene losses, proliferation of short dispersed repeats and introns as well as expansions of intergenic spacers and proteins-coding genes [3,7]. All photosynthetic genes were lost in the lineage leading to the heterotrophic trebouxiophyte *Helicosporidium*, giving rise to an extremely compact genome greatly reduced in both size and gene content [6]. In addition, three expanded genes in chlorophycean green algal cpDNAs were split into two distinct open reading frames (ORFs); this is the case for the *rpoB *genes of *Stigeoclonium*, *Scenedesmus *and *Chlamydomonas*, for *rps2 *of the latter two algae and for *rpoC1 *of *Chlamydomonas *[7]. While chlorophycean green algal cpDNAs were most affected by the abovementioned structural changes, their ulvophyte homologues were altered to an intermediate degree relative to the chlorophycean and *Chlorella *genomes [3,4].

To gain insights into the evolutionary trends of the chloroplast genome in the Trebouxiophyceae and to elucidate the relationships among the various lineages of this class, we undertook the complete sequencing of cpDNA from phototrophic trebouxiophytes occupying lineages distinct from that represented by *Chlorella *and *Helicosporidium *(Chlorellales). We describe here the chloroplast genome sequence of *Leptosira terrestris*, a filamentous alga formally named *Pleurastrum terrestre *[14] and currently thought to belong to the Ctenocladales. Even though this genome resembles its *Chlorella *homologue in missing an IR, it is considerably shuffled in gene order and displays derived features that were previously observed in ulvophyte and/or chlorophycean green algal cpDNAs. Our results highlight the great plasticity of the chloroplast genome in the Trebouxiophyceae.

## Results

### Genomic features

**Figure 1:**
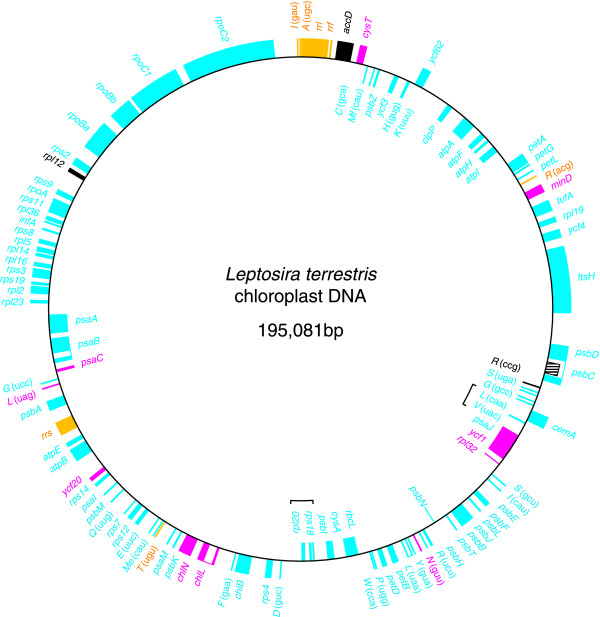
**Gene map of Leptosira cpDNA**. Genes (filled boxes) on the outside of the map are transcribed in a clockwise direction; those on the inside are transcribed counterclockwise. Introns are represented by open boxes and the intron ORF is denoted by a narrow, hatched box. Genes shown in yellow, cyan and magenta map to the IR, LSC and SSC regions of *Mesostigma *cpDNA. Genes and ORFs absent from *Mesostigma *cpDNA are shown in black. Brackets denote the gene clusters shared specifically with *Chlorella *cpDNA. tRNA genes are indicated by the one-letter amino acid code followed by the anticodon in parentheses (*Me*, elongator methionine; *Mf*, initiator methionine).

The *Leptosira *chloroplast genome sequence assembles as a circular molecule of 195,081 bp encoding a total of 106 genes, not counting the intron ORF and the 11 free standing ORFs (Figure 1). All genes are present in single copy. Table 1 compares the general features of *Leptosira *cpDNA with those reported for the eight other chlorophyte cpDNAs completely sequenced to date. With an overall A+T content of 72.7%, the *Leptosira *genome ranks at the third position, after *Helicosporidium *plastid DNA and *Scenedesmus *cpDNA, with respect to the abundance of these bases. The 106 conserved genes, four introns and 11 free standing ORFs of more than 60 codons account for 48.3% of the total genome sequence of this trebouxiophyte, with the introns representing only 2.3% of the sequence. This is the lowest coding density among all examined chlorophyte cpDNAs. Intergenic regions have an average size of 981 bp, a value slightly higher than those observed for *Stigeoclonium *(950 bp) and *Chlamydomonas *(941 bp) cpDNAs. All four introns belong to the group I family.

**Table 1:** General features of *Leptosira *and other chlorophyte cpDNAs

	**Size**							**Introns**	
					
**cpDNA **^a^	**Total**	**IR**	**LSC**	**SSC**	**A+T (%)**	**Coding (%) **^b^	**Genes (no.) **^c^	**I**	**II**
*Nephroselmis *(P)	200,799	46,137	92,126	16,399	57.9	68.7	128	0	0
*Chlorella *(T)	150,613	- ^d^	- ^d^	- ^d^	68.4	60.9	112	3	0
*Leptosira *(T)	195,081	- ^d^	- ^d^	- ^d^	72.7	48.3	106	4	0
*Helicosporidium *(T)	37,454	- ^d^	- ^d^	- ^d^	73.1	94.9	54	1	0
*Oltmannsiellopsis *(U)	151,933	18,510	33,610	81,303	59.5	59.2	104 ^e^	5	0
*Pseudendoclonium *(U)	195,867	6,039	140,914	42,875	62.3	62.3	105	27	0
*Stigeoclonium *(C)	223,902	- ^d^	- ^d^	- ^d^	71.1	55.8	97	16	5
*Scenedesmus *(C)	161,452	12,022	72,440 ^f^	64,968 ^g^	73.1	67.2	96	7	2
*Chlamydomonas *(C)	203,827	22,211	81,307 ^f^	78,088 ^g^	65.5	50.1	94	5	2

^a ^The letter in parentheses indicates the chlorophyte lineage: P, Prasinophyceae; T, Trebouxiophyceae; U, Ulvophyceae; C, Chlorophyceae.

^b ^Conserved genes, unique ORFs and introns were considered as coding sequences.

^c ^Genes present in the IR were counted only once. Unique ORFs and intron ORFs were not taken into account.

^d ^Because *Chlorella, Helicosporidium, Leptosira *and *Stigeoclonium *cpDNAs lack an IR, only the total size is given for each of these genomes.

^e ^The gene repertoire of this alga was erroneously reported to include *trnI*(cau), explaining the difference with the gene number published previously [3, 7, 8].

^f ^This region was designated SC1 by de Cambiaire *et al*. [8] rather than LSC because it differs markedly in gene content relative to the LSC region in *Mesostigma *and *Nephroselmis *cpDNAs.

^g ^This region was designated SC2 by de Cambiaire *et al*. [8] rather than SSC because it differs markedly in gene content relative to the SSC region in *Mesostigma *and *Nephroselmis *cpDNAs.

### Gene content and gene expansions

The chloroplast gene repertoire of *Leptosira *differs from that of *Chlorella *by the absence of three protein-coding genes (*chlI*, *ccsA *and *ycf12*) and three tRNA genes [*trnL*(gag), *trnS*(gga) and *trnT*(ggu)]. Although the latter three genes are missing, the set of 28 tRNA species encoded by *Leptosira *cpDNA is sufficient to read all codons present in this genome. The *trnL*(gag) and *trnT*(ggu) genes are also missing in the two ulvophyte and three chlorophycean green algal chloroplast genomes sequenced so far and *chlI *is absent from all three chlorophycean genomes: however, the *ccsA *and *ycf12 *genes have been retained in all these genomes. Aside from *Leptosira*, the *trnS*(gga) gene has been lost from all UTC algal cpDNAs previously investigated, except *Chlorella *and *Stigeoclonium*.

As in other UTC algal chloroplast genomes, a small fraction of the genes in *Leptosira *cpDNA have expanded coding regions relative to their *Nephroselmis *and streptophyte homologues. Nine genes in the *Leptosira *genome are more than 50% larger than their *Mesostigma *counterparts (*cemA*, *ftsH*, *rpl19*, *rpoA*, *rpoB*, *rpoC1*, *rpoC2*, *ycf1 *and *ycf4*) compared to only three in *Chlorella *cpDNA (*cemA*, *ftsH *and *ycf1*) [3]. In addition, for the latter three expanded genes, we find a more important expansion factor in *Leptosira *than in *Chlorella*.

Like its chlorophycean green algal homologues, the chloroplast *rpoB *gene of *Leptosira *consists of two separate ORFs (*rpoBa *and *rpoBb*) that are not associated with sequences typical of group I or group II introns (Figure 1). As in *Chlamydomonas *and *Scenedesmus *cpDNAs, these ORFs are contiguous in *Leptosira *cpDNA and are separated by stop codons. Reverse transcriptase-PCR analysis failed to identify a genuine transcript encompassing both the *Chlamydomonas rpoBa *and *rpoBb *codingregions [9]; however, distinct transcripts were found to be specific to these ORFs [9]. This result together with the observation that the *Stigeoclonium rpoBa *and *rpoBb *are encoded by different DNA strands and map to separate genomic loci [7] suggest that the two ORFs are transcribed independently. The *Chlamydomonas rpoBa *and *rpoBb *are considered to be functional genes, because no chloroplast-targeted RNA polymerase gene could be identified in the nuclear genome of this alga [15]. The fragmentation of *rpoB *in *Leptosira *cpDNA and its chlorophycean homologues is reminiscent of the well-known case of *rpoC *gene fragmentation in cyanobacteria and plastids (*rpoC1 *and *rpoC2*) [16].

**Figure 2:**
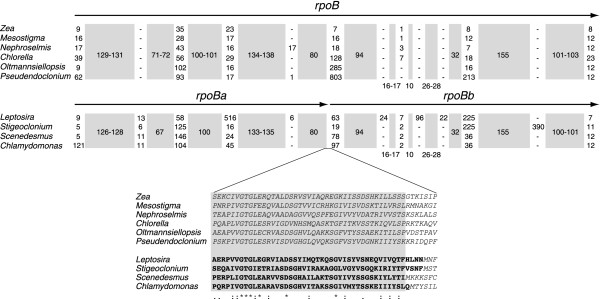
**Comparison of the *Leptosira *RNA polymerase β' subunit with its homologues in other chlorophytes and the streptophytes *Mesostigma *and maize**. The sequences of the *rpoB *gene products were aligned using the fused *rpoBa *and *rpoBb *gene products of *Leptosira*, *Stigeoclonium*, *Scenedesmus *and *Chlamydomonas*. The regions showing significant sequence conservation are denoted by grey boxes, with the numbers inside these boxes referring to amino acids. The numbers of amino acids in the variable regions are also indicated. The lower portion of the figure shows the alignment for the region corresponding to the junction between the *rpoBa *and *rpoBb *gene products. The segment showing significant sequence similarity within this region is represented by the grey box. The C-terminal residues of RpoBa are in bold characters and the N-terminal residues of RpoBb are in italics.

Figure 2 shows the results of our comparative sequence analysis of the translated *rpoB *sequences from *Leptosira*, the seven other photosynthetic chlorophytes whose chloroplast genome has been scrutinized, and two streptophytes. Regions displaying high sequence divergence and significant heterogeneity in size were identified at the two termini and at 11 internal sites. Some of the variable, internal regions represent insertions that are unique to one or more of the taxa examined. For example, the sequences mapping to three sites are present only in *Leptosira*, whereas that corresponding to a fourth site is specific to *Stigeoclonium*. Blast analyses conducted during the course of the present study revealed that the insertion sequence of 390 amino acids at the latter site displays features typical of inteins [17]; this finding was made independently by another laboratory and documented in InBase [17,18] under the accession name She_RPB2. The *rpoB *genes of *Leptosira *and of the three chlorophycean green algae are fragmented at the same site, near the junction of a conserved segment of 80 codons and a highly variable region (ranging from 7 to 803 codons in size) found in all taxa.

The 11 ORFs of more than 60 codons that we identified in intergenic regions of *Leptosira *cpDNA failed to display any similarity with known DNA sequences. All these ORFs differ from the conserved protein-coding genes at the levels of codon usage and their tendency to be richer in A+T.

### Gene distribution between the two DNA strands

**Figure 3:**
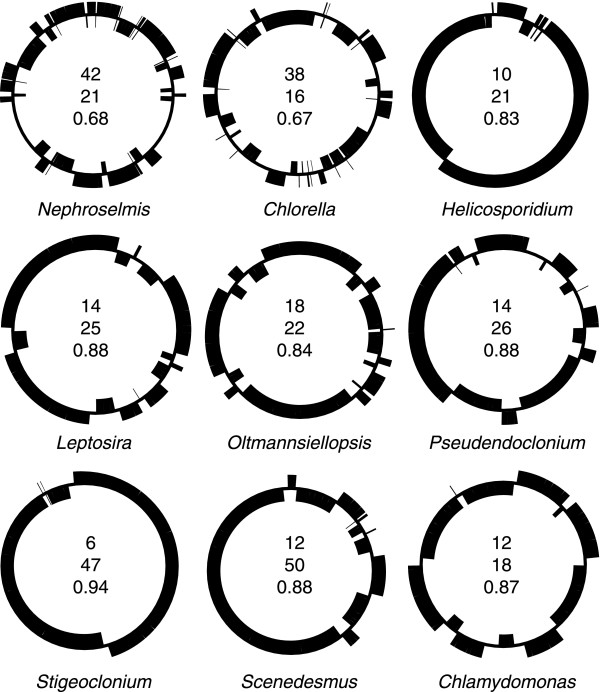
**Distributions of genes on the two DNA strands in *Leptosira *and other green algal cpDNAs**. Black boxes denote blocks of adjacent genes on the same DNA strand. For each genome, the total number of gene blocks (top number), the maximum number of genes observed in a block (middle number) and the sidedness index C_s _(bottom number) are indicated.

With 78 genes occupying one strand and 28 genes the other strand, the gene distribution over the two DNA strands of the *Leptosira *genome is highly biased (Figure 1). While a similar bias has been observed for *Scenedesmus *cpDNA (77 genes on one strand and 26 genes one the other) [8], other completely sequenced chlorophyte cpDNAs display about the same number of genes on the two strands (Figure 3). All 78 *Leptosira *genes found on the same cpDNA strand, except *ycf62 *and *cemA*, are grouped together, forming four separate stretches containing 9 to 24 genes.

The propensity of adjacent genes to be located on the same strand is a property distinguishing all UTC algal cpDNAs, except the *Chlorella *genome, from *Nephroselmis *and streptophyte cpDNAs. The degree to which neighbouring genes are clustered on the same strand is reported in Figure 3 for various chlorophyte chloroplast genomes using the sidedness index (C_s_) of Cui *et al*. [13]. This index was calculated using the formula C_s _= (*n *- *n*_*SB*_)/(*n *- 1), where *n *is the total number of genes in the genome and *n*_*SB *_is the number of sided blocks, *i.e*. the number of blocks containing adjacent genes on the same strand. When C_s _reaches the maximum value of 1, all genes are located on one strand. In Figure 3, it can be seen that the sidedness index of *Leptosira *cpDNA (C_s _= 0.88) is comparable to those of most other UTC algal cpDNAs. However, in contrast to *Stigeoclonium *cpDNA and *Helicosporidium *plastid DNA, analyses of cumulative GC and AT skews indicated that the coding strand bias in the *Leptosira *genome is not associated with a strand bias in base composition.

### Gene order

**Figure 4:**
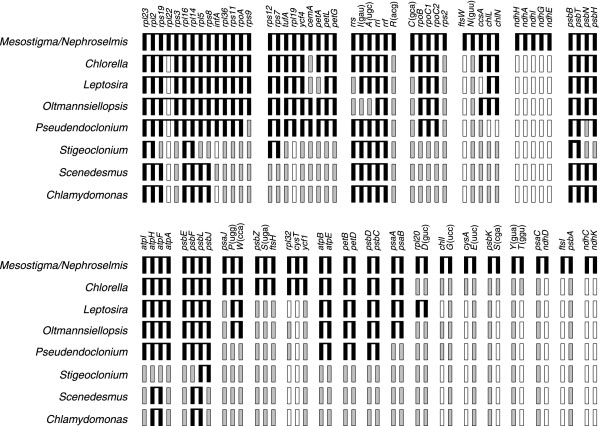
**Conservation of ancestral gene clusters in *Leptosira *and other UTC algal cpDNAs**. Black boxes represent the 89 genes found in the 24 clusters shared by *Mesostigma *and *Nephroselmis *cpDNAs as well as the genes in UTC algal cpDNAs that have retained the same order as those in these ancestral clusters. For each genome, the set of genes making up each of the identified clusters (either an intact or a fragmented ancestral cluster) is shown as black boxes connected by a horizontal line. Black boxes that are contiguous but are unlinked indicate that the corresponding genes are not adjacent on the genome. Grey boxes denote individual genes that have been relocated on the chloroplast genome; open boxes denote genes that have disappeared from the chloroplast genome. Although the *rpl22 *gene is missing from *Nephroselmis *cpDNA, it is shown as belonging to the ribosomal protein cluster equivalent to the contiguous *S10*, *spc *and α operons of *Escherichia coli *because it is present in this cluster in the cpDNAs of *Mesostigma*, streptophytes and algae from other lineages. Note also that the *psbB *cluster of *Pseudendoclonium *differs from the ancestral cluster by the presence of *psbN *on the alternate DNA strand.

Unlike its *Chlorella *homologue, the *Leptosira *chloroplast genome does not reveal any obvious remnant of the ancestral gene partitioning pattern displayed by *Nephroselmis *and most streptophyte cpDNAs (Figure 1). However, the two trebouxiophyte genomes resemble one another with respect to the conservation of ancestral gene clusters (Figure 4). *Leptosira *cpDNA exhibits nine of the 24 ancestral clusters conserved between *Mesostigma *and *Nephroselmis *cpDNAs as well as the remains of five other clusters; altogether, these conserved clusters encode 53 genes. In *Chlorella*, 62 genes are found to be part of 11 intact and four partially conserved, ancestral clusters. Breakage of three of the intact clusters present in *Chlorella *cpDNA, further fragmentation of the four partially conserved clusters in this alga and specific retention of an ancestral cluster [*rpl20*-*trnD*(guc] absent from the *Chlorella *and other UTC cpDNAs explain the main differences observed in the *Leptosira *genome. Note that these rearrangement events involved the disruption of the highly conserved rRNA operon [*rrs-trnI*(gau)*-trnA*(ugc)*-rrl-rrf*] downstream of *rrs *and the relocalization of this gene 62 kb away from the remaining portion of the operon.

Only two derived gene clusters, *trnV*(uac)*-trnL*(caa) and *rpl20-rps18*, are shared specifically between *Leptosira *and *Chlorella *cpDNAs. The *rpl20-rps18 *gene pair is also conserved in the ulvophytes *Oltmannsiellopsis *and *Pseudendoclonium*.

To compare the degrees of similarity in gene order between the *Leptosira *and other green algal cpDNAs, we also estimated the minimal number of gene permutations that would be required to convert the gene order of a given genome to that of another genome. More specifically, we examined the orders of the 86 genes common to the cpDNAs of *Leptosira*, *Chlorella*, *Nephroselmis*, *Oltmannsiellopsis*, *Pseudendoclonium, Stigeoclonium, Scenedesmus *and *Chlamydomonas *using GRIMM [19]. We found that the *Leptosira *and *Chlorella *genomes differ by as many as 53 inversions. As shown in Table 2, this level of gene rearrangements is identical to that found for the cpDNAs of the ulvophytes *Oltmannsiellopsis *and *Pseudendoclonium *but lower than those obtained for the chlorophycean algae *Stigeoclonium*, *Scenedesmus *and *Chlamydomonas *(59–83 inversions). Furthermore, our results showed that the *Nephroselmis*, ulvophyte and chlorophycean genomes, except *Stigeoclonium *cpDNA, display more similarity in gene order with the *Chlorella *genome than with *Leptosira *cpDNA.

**Table 2:** Minimal numbers of inversions estimated in pairwise comparisons of gene order in chlorophyte cpDNAs

	**Number of inversions **^a^						
	
**Compared cpDNAs**	***Chlorella***	***Leptosira***	***Oltmannsiellopsis***	***Pseudendoclonium***	***Stigeoclonium***	***Scenedesmus***	***Chlamydomonas***
*Nephroselmis*	46	51	54	54	79	73	72
*Chlorella*		53	50	50	78	73	71
*Leptosira*			55	58	77	74	72
*Oltmannsiellopsis*				53	81	75	74
*Pseudendoclonium*					77	74	73
*Stigeoclonium*						78	83
*Scenedesmus*							59

^a ^Numbers of gene permutations by inversions were computed using GRIMM [19].

### Intron content

The four group I introns in *Leptosira *cpDNA interrupt *chlL*, *psaB*, *psbC *and *trnL*(uaa). They fall within two different subgroups (IA1 and IC3), with the IA1 subgroup including the three introns found in protein-coding genes. All four introns, except that present in *psbC*, are positionally and structurally homologous to previously reported introns in chlorophyte cpDNAs (Table 3). In this context, it is worth noting that *Leptosira *and *Chlorella *cpDNAs share homologous introns in *trnL*(uaa) and *chlL*. The *psbC *intron is unique to *Leptosira *and displays an ORF. The predicted protein of 415 amino acids specified by this ORF is related to the LAGLIDADG endonucleases and contains two copies of this motif.

**Table 3:** Introns in *Leptosira *cpDNA and homologous introns at identical gene locations in other chlorophyte cpDNAs

***Leptosira *intron**			**Homologous introns**	
**Designation**	**Subgroup **^a^	**ORF location **^b^	**Green alga **^c^**/Intron number **^d^	**Accession no.**

Lt.*trnL*(uaa)	IC3	-	*Bryopsis plumosa *(U)	GenBank:M61159
			*Chlorella vulgaris *(T)	GenBank:NC_001865
			*Scenedesmus obliquus *(C)	GenBank:M90641
Lt.*chlL*	IA1	-	*Chlorella vulgaris *(T)	GenBank:NC_001865
Lt.*psaB*	IA1	-	*Chlamydomonas moewusii *(C)	GenBank:M90641
			*Scenedesmus obliquus *(C)	GenBank:M90641
			*Stigeoclonium helveticum *i2 (C)	Genbank:DQ630521
Lt.*psbC*	IA1	L9.3	-	-

^a ^Group I introns were classified according to Michel and Westhof [48].

^b ^L followed by a number refers to the loop extending the base-paired region identified by the number.

^c ^The letter in parentheses denotes the chlorophyte lineage comprising the green alga indicated: U, Ulvophyceae; T, Trebouxiophyceae; C, Chlorophyceae.

^d ^The intron number is given when more than one intron is present.

### Repeated sequences

**Figure 5:**
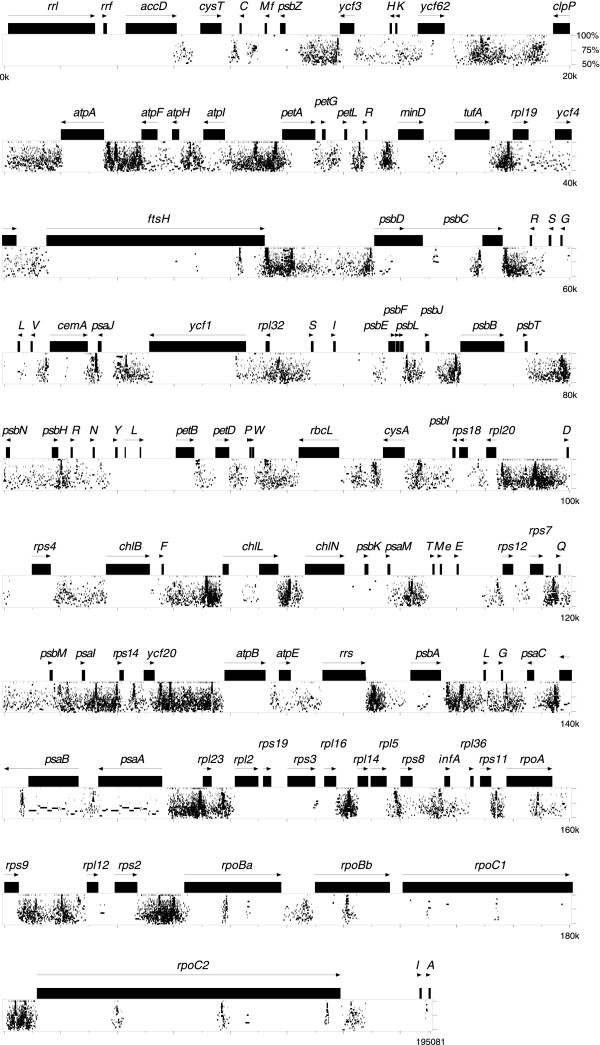
**PipMaker analysis of *Leptosira *cpDNA**. The genome sequence was aligned against itself. Similarities between aligned regions are shown as average percent identity (between 50% and 100% identity). Genes and their polarities are denoted by horizontal arrows and coding sequences are represented by filled boxes.

Comparison of the *Leptosira *cpDNA sequence against itself reveals that short repeats are present in many intergenic regions as well as in some introns and genes (predominantly expanded genes) (Figure 5). Repeats represent 5.1% of the total genome sequence and 8.5% of the total size of the intergenic regions. The relative abundance of these elements is therefore lower than in *Chlorella *cpDNA (7.8% of total genome and 14.9% of intergenic regions) but is comparable to those observed in *Scenedesmus *and *Pseudendoclonium *cpDNAs (see Table 2 in [20]). In all other completely sequenced cpDNAs of photosynthetic UTC algae, short repeats account for more than 10% of the genome. The longest repeated sequence in *Leptosira *cpDNA is 194 bp in size, whereas the maximal size of the *Chlorella *repeats is two-fold smaller (84 bp).

The most abundant repeated sequences in the *Leptosira *genome consist of dispersed repeats. Analysis of these repeats revealed two distinct groups of repeat units: repeat unit A with sequences of 25 bp (TTYAYCTGGGCAGGGAGATYYGRTC) and repeat unit B with sequences of 18 bp (CRGTWWATAAATCWWWGA). Each group of repeats features variants that differ slightly in primary sequence. Altogether, the 81 copies of repeat unit A and the 74 copies of repeat unit B represent 1.7% of the *Leptosira *genome sequence. In term of localization, a close relationship exists between repeat units A and B. Copies of repeat unit A are frequently found to be contiguous with the reverse complement of an almost identical sequence, creating imperfect palindromes. Copies of repeat unit B, in turn, are usually associated with such palindromes to generate larger palindromes of the type B-A-A_rev_-B_rev_, where rev stands for reverse complement. No repeats identical to the *Leptosira *repeat units A and B were detected in any other completely sequenced UTC algal cpDNA.

## Discussion

### Multiple losses of the IR during the evolution of trebouxiophytes

As in *Chlorella vulgaris *cpDNA and *Helicosporidium *plastid DNA, we found that a rDNA-encoding IR is missing from the *Leptosira *chloroplast genome. Despite the absence of the IR in these three trebouxiophyte DNAs, there is little doubt that the chloroplast genome from the common ancestor of all trebouxiophytes featured a quadripartite structure very similar to that found in streptophytes and the prasinophyte *Nephroselmis*. This inference is supported by two separate observations. First, the partially sequenced chloroplast genome of *Chlorella ellipsoidea*, a representative of the trebouxiophyte order Prasiolales, displays a large IR, even though the latter region is atypical in containing a disrupted rDNA operon [21]. Second, the IR-lacking *Chlorella vulgaris *cpDNA retains not only a remnant of an IR in the form of a pseudo *rrs *gene [5] but also the ancestral partitioning of genes displayed by prasinophyte cpDNA [3,10].

Although the divergence order of the various monophyletic groups recognized in the Trebouxiophyceae remains ambiguous, the currently available phylogenetic data suggest that at least two distinct events of IR loss account for the disappearance of the IR in the three sequenced trebouxiophyte chloroplast genomes. The Trebouxiophyceae is a morphologically diverse assemblage that includes lichen phycobionts such as *Trebouxia*, free-living planktonic or terrestrial species, secondarily nonphotosynthetic coccoid algae and picoplanktonic coccoids [22,23]. At least five distinct monophyletic lineages are recovered with 18S rDNA data [24-27], four of which correspond to the Trebouxiales, Microthamniales, Prasiolales and Chlorellales. Members of the Chlorellales, which include both *Chlorella vulgaris *and *Helicosporidium*, are consistently identified with high bootstrap support as the earliest-diverging branch of the Trebouxiophyceae, but the interrelationships among the remaining trebouxiophyte lineages remain ambiguous. This tree topology supports the view that the IR was lost independently in the Chlorellales and in the lineage leading to *Leptosira*. Obviously, for the Chlorellales, a single loss event is the most parsimonious explanation for the absence of the IR in *Chlorella vulgaris *cpDNA and *Helicosporidium *plastid DNA. To distinguish this scenario from the alternative hypothesis involving two independent losses, additional members of the Chlorellales will need to be surveyed for the presence/absence of this repeat.

In the light of previous reports indicating that loss of the chloroplast IR occurred relatively frequently during the evolution of the Viridiplantae, our inference that the IR was lost independently on at least two separate occasions in the Trebouxiophyceae does not imply that the quadripartite structure is less unstable in this algal group than in others. Aside from the Trebouxiophyceae, chloroplast genomes that experienced complete or almost-complete loss of the IR have been documented for the chlorophyte classes Ulvophyceae [28] and Chlorophyceae [7], for the charophycean lineage leading to the Zygnematales [29,30] and for a number of land plants, including conifers and six tribes of legumes [31,32]. Losses of the IR in conifers and legumes occurred independently and differed in the extent of the IR sequence lost, in the gene content of the IR prior to loss, and in the copy of the IR that was deleted [32]. The site of deletion in pea cpDNA was found to exhibit duplicated gene fragments, but no simple mechanism involving recombination between these repeats could be postulated to account for the IR loss [33]. In the present study, it was not possible to elaborate evolutionary models for the IR losses sustained by green algal cpDNAs, because the highly variable gene organization found in these genomes precluded inferences of gene order in ancestral IR-containing cpDNAs. Chloroplast genome sequences from more trebouxiophytes will thus be required to gain deeper insight into how the IR was deleted.

### Similar evolutionary forces may have shaped the IR-lacking *Leptosira *and *Stigeoclonium *chloroplast genomes

We found that the *Leptosira *chloroplast genome differs considerably from its *Chlorella vulgaris *counterpart not only in gene order, but also in gene density, gene distribution between the two DNA strands and structure of some protein-coding genes. The important changes in gene order (Table 2) and in conservation level of ancestral gene clusters (Figure 4) observed for these trebouxiophyte cpDNAs are not surprising, given that IR loss is generally correlated with gene rearrangements [11,12]. On the basis of this correlation, it has been hypothesized that IR loss enhances the frequency of intramolecular recombination between short dispersed repeats [31]. In this context, it is worth mentioning that no short dispersed repeats = 30 bp with over 90% sequence identity are shared between the intergenic regions of *Leptosira *and *Chlorella vulgaris *cpDNAs, suggesting that these elements evolved independently in these two trebouxiophyte lineages. The fact that the *Chlorella vulgaris *genome displays a more ancestral gene order than its *Leptosira *homologue might be due to a more recent loss of the IR and/or a more recent proliferation of short repeats in the *Chlorella vulgaris *lineage.

Most intriguingly, the *Leptosira *chloroplast genome exhibits derived traits that are reminiscent of the evolutionary pattern observed for ulvophyte and/or chlorophycean cpDNAs. These derived traits were identified in the course of analyzing the following genomic features: (1) gene distribution over the two DNA strands, (2) gene density and (3) expansion and structure of protein-coding genes. The *Leptosira *chloroplast genes display a highly biased and asymmetrical distribution pattern over the two DNA strands, which most closely matches that observed for the chloroplast genome of the chlorophycean green alga *Scenedesmus *(Figure 3). The strong propensity of adjacent genes to be located on the same DNA strand in *Leptosira *cpDNA also mirrors the gene distribution patterns found in the chloroplast/plastid genomes of the two other chlorophyceans investigated (*Stigeoclonium *and *Chlamydomonas*), the ulvophytes *Oltmannsiellopsis *and *Pseudendoclonium *and the trebouxiophyte *Helicosporidium*. With regard to gene density, *Leptosira *cpDNA is currently known to be the most loosely packed chlorophyte genome (Table 1), followed by the *Chlamydomonas *and *Stigeoclonium *cpDNAs. The chloroplast genes exhibiting expanded coding regions relative to their *Nephroselmis *and streptophyte homologues are three-times more abundant in *Leptosira *than in *Chlorella vulgaris*, with the *Leptosira *set of nine expanded genes being more similar to those found in ulvophyte genomes with respect to coding content. In contrast to the conventional structure observed for *rpoB *in *Chlorella vulgaris *and ulvophytes, the *Leptosira rpoB *gene is fractured at the same site as that found for the fragmented genes of the three analyzed chlorophycean green algae (Figure 2). Therefore, two separate events of gene fragmentation, one occurring in the *Leptosira *lineage and the other before the emergence of the three chlorophycean groups examined so far, must be postulated to account for the distribution of the split *rpoB *structure among UTC algae.

From the similarities described above, it is tempting to propose that the same evolutionary forces shaped the IR-lacking chloroplast genomes in trebouxiophyte and chlorophycean lineages. However, considering the extraordinary fluidity of the chloroplast genome structure in the Chlorophyceae and the fact that no IR-containing chloroplast genomes from close relatives of *Leptosira *and *Stigeoclonium *have been investigated, it remains uncertain whether the common trends identified here are directly linked with the convergent events of IR loss that occurred in these chlorophyte lineages. For the Streptophyta, more specifically the zygnematalean lineages leading to *Staurastrum *and *Zygnema*, there exists convincing evidence that IR loss from the chloroplast genome was correlated with the expansion of intergenic regions and extensive gene rearrangements [30]. Indeed, the low degree of compaction, the highly scrambled gene order and the numerous disrupted ancestral clusters observed in the *Staurastrum *and *Zygnema *genomes contrast sharply with the short intergenic spacers and with the extraordinary conservation of gene order and ancestral clusters exhibited by all their homologues in other streptophyte lineages.

## Conclusion

The numerous derived features that we report here for the IR-lacking *Leptosira *chloroplast genome contrast sharply with the pronounced degree of ancestral features displayed by *Chlorella vulgaris *cpDNA, a trebouxiophyte genome also missing a rDNA-encoding IR. The close resemblance of the *Leptosira *genome with its ulvophyte and/or chlorophycean homologues with respect to the pattern of gene distribution, gene density and structure of protein-coding genes was also an unanticipated finding. On the basis of the current knowledge regarding the phylogeny of trebouxiophytes and the distribution of the presence/absence of the IR in the chloroplast genome, we conclude that the IR was lost independently in the Chorellales and the *Leptosira *lineage. The intriguing similarities between the derived features exhibited by the *Leptosira *chloroplast genome and those of its chlorophycean counterparts might suggest that the same evolutionary forces shaped the IR-lacking chloroplast genomes in the *Leptosira *and chlorophycean lineages. To test this hypothesis and better understand the dynamics of IR loss, IR-containing chloroplast genomes from close relatives of *Leptosira *and *Stigeoclonium *will need to be investigated.

## Methods

### Isolation and sequencing of *Leptosira *cpDNA

*Leptosira terrestris *(formally *Pleurastrum terrestre *Fritsch et John) was obtained from the University of Texas Algal Culture collection (UTEX 333) and grown in modified Volvox medium [34] under 12 h light-dark cycles. An A+T rich fraction containing cpDNA was isolated and sequenced as previously described [35]. Sequences were assembled and edited with SEQUENCHER 4.2 (Gene Codes Corporation, Ann Harbor, MI). The fully annotated genome sequence has been deposited in [GenBank:EF506945].

### Sequence analyses

Genes were identified by BLAST searches [36] against the nonredundant database of the National Center for Biotechnology Information server (NCBI) [37]. Positions of ORFs and protein coding genes were determined using ORFFINDER at NCBI, programs of the GCG Wisconsin package (version 10.3) (Accelrys, San Diego, CA, USA) and applications from the EMBOSS version 2.9.0 package [38]. Gene coding for tRNAs were localized with tRNAscan-SE 1.23 [39]. The RpoB sequences were aligned using ClustalW 1.82 [40]. Repeated sequences were identified with PipMaker [41] and REPuter 2.74 [42]. Repeats were sorted with REPEATFINDER [43] and the retrieved classification was refined manually. Numbers of SDR units were determined with FINDPATTERNS of the GCG Wisconsin package version 10.3. The total length of genome sequences containing repeated elements was estimated with RepeatMasker [44] running under the WU-BLAST 2.0 search engine [45]. Separate files containing the concatenated sequences of the intergenic regions of *Leptosira *and *Chlorella *cpDNAs were produced to search for the presence of shared repeated elements = 30 bp with up to 10% mismatches using the *-d -p -l 30 -e 3 -seedlength 10 -q -v *options of V *match *[46]. The results of this analysis were visualized using GenAlyzer 0.81b [47].

### Analyses of genome rearrangements

The GRIMM web server [19] was used to infer the minimal number of gene permutations by inversions in pairwise comparisons of chloroplast genomes. For these analyses, genes within one of the two copies of the IR were excluded from the data set and only the genes common to all compared genomes were analyzed. The data set used in the comparative analyses reported in Table 2 contained 86 genes; the three exons of the trans-spliced *psaA *and *rbcL *genes, the two exons of the trans-spliced *psaC *and *petD *genes, as well as the *rpoBa *and *rpoBb *genes, were coded as distinct fragments (for a total of 93 loci).

## Authors' contributions

JCdC participated in the conception of this study, carried out part of the genome sequencing, performed all sequence analyses, annotated the genome, generated the tables and figures and drafted the manuscript. CO participated in the sequencing and contributed to the assembly of the genome sequence. CL and MT conceived and supervised the study, contributed to the interpretation of the data and prepared the manuscript. All authors read and approved the final manuscript.
